# CD161 Defines a Transcriptional and Functional Phenotype across Distinct Human T Cell Lineages

**DOI:** 10.1016/j.celrep.2014.09.045

**Published:** 2014-10-23

**Authors:** Joannah R. Fergusson, Kira E. Smith, Vicki M. Fleming, Neil Rajoriya, Evan W. Newell, Ruth Simmons, Emanuele Marchi, Sophia Björkander, Yu-Hoi Kang, Leo Swadling, Ayako Kurioka, Natasha Sahgal, Helen Lockstone, Dilair Baban, Gordon J. Freeman, Eva Sverremark-Ekström, Mark M. Davis, Miles P. Davenport, Vanessa Venturi, James E. Ussher, Christian B. Willberg, Paul Klenerman

**Affiliations:** 1 Peter Medawar Building for Pathogen Research, University of Oxford, Oxford OX1 3SY, UK; 2 Department of Microbiology and Infectious Disease, Oxford University Hospitals NHS Trust, Oxford OX3 9DU, UK; 3 Department of Microbiology and Immunology, Stanford University, Stanford, CA 94305, USA; 4 Agency for Science, Technology and Research (A^∗^STAR), Singapore Immunology Network (SIgN), Singapore 138632, Singapore; 5 Department of Molecular Biosciences, The Wenner-Gren Institute, Stockholm University, 106 91 Stockholm, Sweden; 6 Bioinformatics and Statistical Genetics Core, Wellcome Trust Centre for Human Genetics, University of Oxford, Oxford OX3 7BN, UK; 7 Dana Farber Cancer Institute, Harvard Medical School, Boston, MA 02215, USA; 8 Department of Haematology, Prince of Wales Hospital, Kensington, NSW NS2 2052, Australia; 9 Department of Microbiology and Immunology, University of Otago, Dunedin 9054, New Zealand; 10 NIHR Oxford Biomedical Research Centre, John Radcliffe Hospital, Oxford OX3 9TU, UK

## Abstract

The C-type lectin CD161 is expressed by a large proportion of human T lymphocytes of all lineages, including a population known as mucosal-associated invariant T (MAIT) cells. To understand whether different T cell subsets expressing CD161 have similar properties, we examined these populations in parallel using mass cytometry and mRNA microarray approaches. The analysis identified a conserved CD161++/MAIT cell transcriptional signature enriched in CD161+CD8+ T cells, which can be extended to CD161+ CD4+ and CD161+TCRγδ+ T cells. Furthermore, this led to the identification of a shared innate-like, TCR-independent response to interleukin (IL)-12 plus IL-18 by different CD161-expressing T cell populations. This response was independent of regulation by CD161, which acted as a costimulatory molecule in the context of T cell receptor stimulation. Expression of CD161 hence identifies a transcriptional and functional phenotype, shared across human T lymphocytes and independent of both T cell receptor (TCR) expression and cell lineage.

## Highlights

• CD161 expression defines specific T cell subsets, including CD8+, CD4+, and TCRγδ+
• CD161-expressing lymphocytes possess a conserved transcriptional signature
• CD161-expressing lymphocytes display a shared innate response to IL-12+18
• CD161 can act as a costimulatory receptor

## Graphical Abstract

**Figure:**
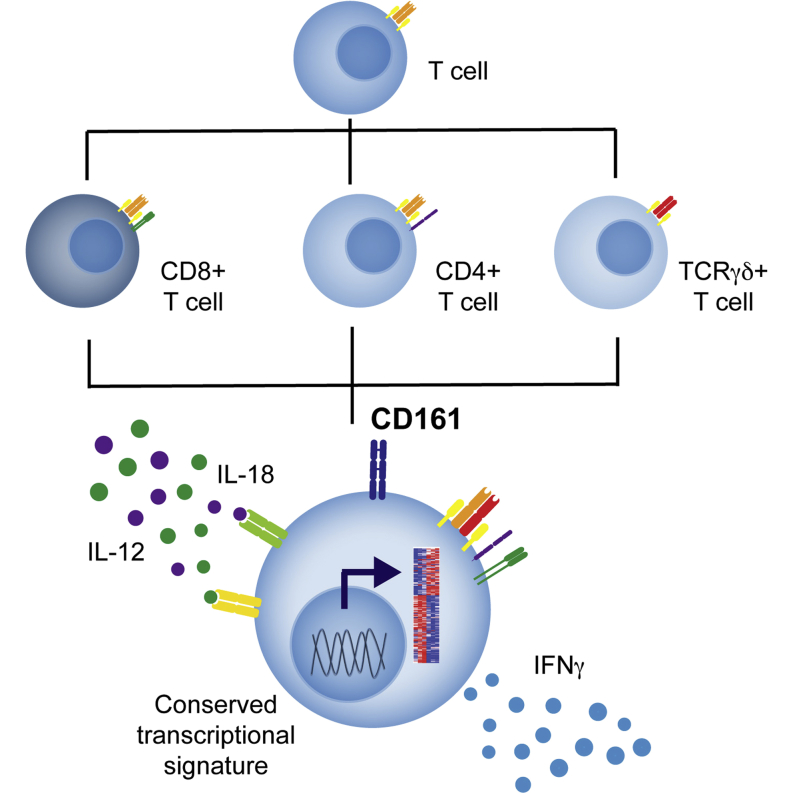

## Teaser

T lymphocytes are conventionally divided into subsets based on expression of coreceptors, cytokines, and surface molecules. Using CyTOF and mRNA microarray analysis, Fergusson et al. identify T lymphocytes that express the C-type lectin CD161 to share a transcriptional profile and innate function across these previously defined subsets.

## Introduction

T lymphocytes form a major arm of the adaptive response, with somatic recombination of the T cell receptor (TCR) enabling recognition of a wide variety of antigens, coupled with the ability to form immunological memory. Although developing from a common thymic precursor, T lymphocytes may develop to express a TCR composed of either γδ chains or, more conventionally in humans, of αβ. TCRαβ+ T cells subsequently develop to be either CD8+ or CD4+, displaying distinct functions and restricted by major histocompatibility complex (MHC) class I or II molecules, respectively. These subsets display further subdivisions, with differentiation of CD4+ T cells into defined helper cell subsets characterized by unique cytokine production, transcription factor expression, and surface phenotype. For example, the more recently defined Th17 subset is characterized by secretion of interleukin (IL)-17, the master transcription factor RORγt (Annunziato et al., 2007), and expression of the C-type lectin CD161 (Cosmi et al., 2008).

Expression of CD161 is not restricted to CD4+ Th17 cells, however. Originally a marker of natural killer (NK) cells (Lanier et al., 1994, Yokoyama and Seaman, 1993), T cell expression of CD161 was identified two decades ago on both CD4+ and CD8+ (Lanier et al., 1994), and later on TCRγδ+ (Battistini et al., 1997), T cells. Indeed, a quarter of both TCRαβ+ T cells (Lanier et al., 1994) and TCRγδ+ T cells (Battistini et al., 1997) express this C-type lectin, and thus CD161 is expressed by a large proportion of human T cells. Within CD8+ T cells, two populations are evident, expressing either intermediate or high levels of CD161 (CD161+ or CD161++; Takahashi et al., 2006), with the latter shown to consist mainly of mucosal-associated invariant T (MAIT) cells (Martin et al., 2009, Ussher et al., 2014).

MAIT cells are a family of innate-like human T cells that display a somatically recombined yet semi-invariant TCR, composed of the TCR α chain Vα7.2-Jα33/12/20 (Reantragoon et al., 2013, Tilloy et al., 1999) paired with a biased Vβ repertoire (Reantragoon et al., 2013, Walker et al., 2012). Expression of this TCR restricts MAIT cells to the MHC class Ib antigen-presenting molecule MR1, which presents riboflavin precursors (Corbett et al., 2014, Kjer-Nielsen et al., 2012) produced by a variety of bacteria to activate MAIT cells. Whereas originally identified within the double-negative T cell fraction (Porcelli et al., 1993), approximately 90% of MAIT cells in humans are CD8+ (either CD8αα or αβ; Walker et al., 2012), although a minor fraction of CD4+ MAIT cells also exists (Reantragoon et al., 2013). Yet, independently of coreceptor expression, all MAIT cells are identified by high expression of CD161 (Dusseaux et al., 2011, Martin et al., 2009).

CD161 is a homodimeric C-type lectin, which represents the single human ortholog of the family of *NKRP1* genes in rodents (Lanier et al., 1994), and thus study of MAIT and CD161-expressing T cells is currently restricted to the human system. Murine NKRP1 receptors recognize non-MHC ligands of the C-type lectin-related (Clr) family, encoded by genes interspersed within the *NKRP1* genes themselves (Iizuka et al., 2003, Plougastel et al., 2001). Similarly, CD161 binds the human ortholog of Clr-b, known as lectin-like transcript 1 (LLT1) (Aldemir et al., 2005, Rosen et al., 2005). Whereas the outcome of CD161 ligation on NK cells is generally accepted to be inhibitory (Aldemir et al., 2005, Lanier et al., 1994, Rosen et al., 2005), the effect on T cells is less clear, with reports of both costimulatory (Aldemir et al., 2005, Exley et al., 1998) and inhibitory (Le Bourhis et al., 2013, Rosen et al., 2008) effects.

Both CD161++ MAIT cells and CD161+CD4+ T cells display a type 17 phenotype (Billerbeck et al., 2010, Cosmi et al., 2008, Dusseaux et al., 2011). This phenotype appears preprogrammed, with precursors of both MAIT and Th17 cells identified within umbilical cord blood by expression of CD161 (Cosmi et al., 2008, Walker et al., 2012). Indeed, a highly significant correlation in gene expression in CD161++CD8+ T cells between cord blood and adults was demonstrated, despite only a minor proportion of CD161++CD8+ T cells expressing the MAIT cell TCR at birth (Walker et al., 2012). Furthermore, CD161 has previously been shown to identify T cells with the potential to produce IL-17 (Maggi et al., 2010). Therefore, we asked whether expression of CD161 marked cells with a shared phenotypic or transcriptional profile, both within and across previously defined T cell subsets, and further whether this corresponded to a specific shared function between these otherwise disparate cell types.

## Results

### CD161++ CD8+ T Cells, Including Both Vα7.2+ MAIT and Vα7.2− Populations, Share a Common Phenotype

**Figure 1:**
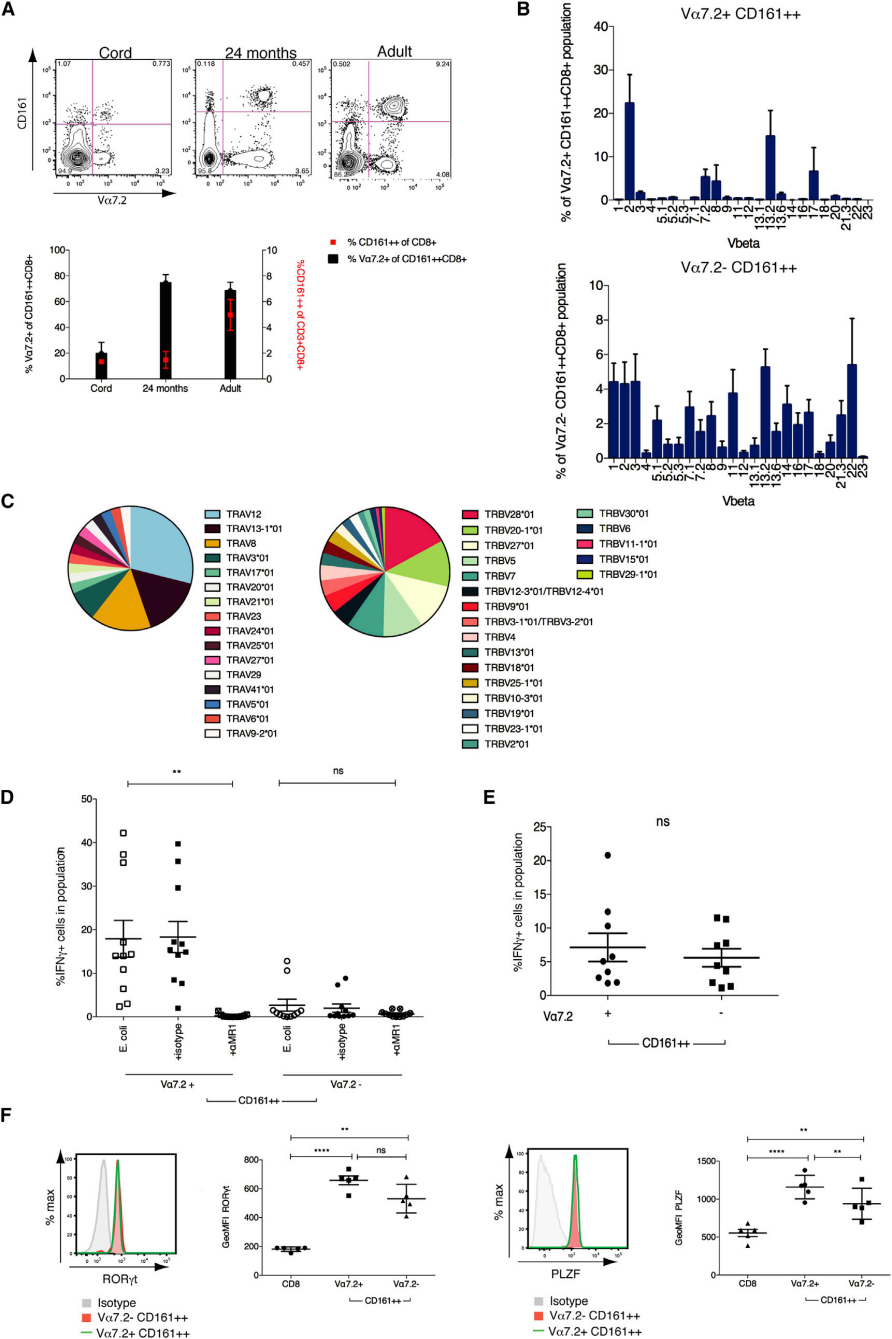
Polyclonal Vα7.2−CD161++ CD8+ T Cells Are Not Activated in an MR1-Dependent Manner but, like MAIT Cells, Respond to Stimulation by IL-12+IL-18 (A) Percentage Vα7.2+ of CD161++CD8+ T cells (histogram) and percentage CD161++ of CD8+ T cells (red point) in cord (n = 3), 24 month (n = 8), and adult blood (n = 8). Representative flow cytometry plots shown above. (B) Comparison of TCR Vβ usage by Vα7.2+ and Vα7.2− CD161++ CD8+ T cells using a TCR Vβ antibody panel. (C) Proportional Vα (38 sequences) and Vβ (129 sequences) usage by Vα7.2− CD161++CD8+ T cells as determined by single-cell TCR sequencing (n = 3). (D) Percentage of IFNγ expression by Vα7.2+ or Vα7.2− CD161++ CD8+ T cell subsets cocultured with THP1 cells exposed to *E. coli* in the presence or absence of anti-MR1 (10 μg/ml) or corresponding isotype control (n = 11). ^∗∗^p < 0.01; one-way ANOVA with Dunnett’s multiple comparisons test compared to *E. coli* alone. (E) Percentage of IFNγ expression by sorted Vα7.2+ or Vα7.2− CD161++CD8+ T cells incubated overnight with IL-12 + IL-18 at 50 ng/ml (n = 9). ns, not significant by paired t test. (F) Transcription factor expression as determined by flow cytometry. Graphs show GeoMFI of RORγt (left) and PLZF (right) by conventional CD8+ T cells (CD3+CD8+ cells excluding CD161++) and Vα7.2+ or Vα7.2− CD161++ CD8+ T cells (n = 5). ^∗∗∗∗^p < 0.0001; ^∗∗^p < 0.01; one-way ANOVA with Tukey’s multiple comparison test. Representative histograms show isotype (gray), Vα7.2+ (green line), and Vα7.2− (red) for each transcription factor. All data are represented as mean ± SEM. See also Figure S1.

MAIT cells express an invariant TCR α chain (Vα7.2) together with high levels of CD161 (Dusseaux et al., 2011) and predominantly express the CD8 coreceptor (>90%; Walker et al., 2012). Whereas Vα7.2+ MAIT cells represent the vast majority of the CD161++ CD8+ T cell population (<95%) in adult blood, a population of CD161++CD8+ T cells that are Vα7.2− is also evident (Figure 1A), comprising an average of 11.41% (±6.99%) of CD161++CD8+ T cells in healthy adults. We first investigated the identity of this additional CD161++CD8+ T cell population in relation to MAIT cells. In cord blood, where CD161++CD8+ T cells are already preprogrammed to a MAIT-like, type-17 phenotype (Walker et al., 2012), this Vα7.2− population comprised the major proportion of CD161++CD8+ T cells (84.69% ± 8.44%). After birth, Vα7.2+ MAIT cell expansion drives the MAIT cell domination of the CD161++ population (Gold et al., 2013, Leeansyah et al., 2014), and a corresponding reduction in the proportion of Vα7.2− cells was seen. This was evident as early as 24 months, where the MAIT cell population represented nearly 75% of the CD161++CD8+ T cell population (Figure 1A).

In addition to invariant expression of Vα7.2, MAIT cells display semi-invariant TCR β usage (Figure 1B) with predominant use of Vβ2 and Vβ13.2, as previously described (Reantragoon et al., 2013, Walker et al., 2012). In contrast, a panel of Vβ antibodies revealed nondiscriminate Vβ usage by Vα7.2− CD161++CD8+ T cells. We further investigated the TCR repertoire of this population by TCR sequencing, performed on single cells, as described (Wang et al., 2012). This confirmed Vβ usage by Vα7.2− CD161++CD8+ T cells to be polyclonal. Furthermore, polyclonality within α chain usage was also evident, with expression of a variety of α chains both within and between donors (Figure 1C).

The semi-invariant MAIT cell TCR recognizes bacterially derived riboflavin precursors presented by the antigen-presenting molecule MR1 (Corbett et al., 2014, Kjer-Nielsen et al., 2012). MHC-tetramer-positive populations against various viral epitopes are rarely CD161++ (J.R.F., unpublished data). Therefore, to establish the MR1 reactivity of Vα7.2− CD161++CD8+ T cells, we used a 5 hr coculture assay in which we have previously described MAIT cells to be activated via the TCR to produce interferon γ (IFNγ) (Ussher et al., 2014). Here, THP1s were incubated with fixed *Escherichia coli* (*E. coli*) overnight and then cultured with sort-purified Vα7.2+ or Vα7.2− CD161++CD8+ T cells. As expected, CD161++Vα7.2+ MAIT cells produced IFNγ in response to THP1s + *E. coli*, and these responses were blocked by addition of anti-MR1 (Figure 1D). Whereas in two donors, a limited MR1-mediated response by Vα7.2− CD161++CD8+ T cells to *E.coli* was apparent, the majority of donors did not respond to bacteria. No response was observed in sorted conventional (MHC-restricted) CD161+CD8+ T cells (data not shown). This was reflected in 24-month-old donors where no response was observed from Vα7.2− CD161++CD8+ T cells when peripheral blood mononuclear cells (PBMCs) were incubated for 5 hr with *E. coli*-loaded THP1s (Figure S1).

In addition to MR1-mediated stimulation through the TCR, MAIT cells are also activated by a combination of IL-12+IL-18 in a TCR-independent, innate manner, a characteristic that was shared by other CD161++CD8+ T cells (Ussher et al., 2014). Gating of both CD161++CD8+ T cell populations in adult, 24-month-old, and cord blood donors again showed that both Vα7.2+ and Vα7.2− portions were capable of IFNγ production (Figure S1). We confirmed this in sorted adult populations, where both Vα7.2+ and Vα7.2− CD161++CD8+ T cells produced IFNγ in response to cytokine stimulation (Figure 1E), indicating this as a feature of CD161++CD8+ T cells as a whole.

We next asked whether Vα7.2− CD161++CD8+ T cells shared the characteristic transcription factor expression of MAIT cells, namely expression of RORγt, associated with the type-17 profile of MAIT cells (Billerbeck et al., 2010, Dusseaux et al., 2011) and promyelocytic leukemia zinc finger (PLZF), related to the innate-like effector differentiation of MAIT and NK T (NKT) cells (Savage et al., 2008). Expression of these transcription factors by Vα7.2− CD161++CD8+ was significantly (p < 0.01) higher than in conventional CD8+ T cells but expressed on average at slightly lower levels than MAIT cells (Figure 1F). Vα7.2− CD161++CD8+ T cells also principally displayed an effector memory (CD62L−CD45RA−) phenotype, as seen in Vα7.2+ MAIT cells (Figure S1).

Together, these results illustrate that, despite different TCR expression and restriction, both Vα7.2+ MAIT and Vα7.2− CD161++CD8+ T cells share a preprogrammed phenotype and an innate ability to respond to cytokine stimulation and can therefore be viewed as a common population related through high expression of CD161.

### Analysis of CD161-Expressing CD8+ T Cells by CyTOF

**Figure 2:**
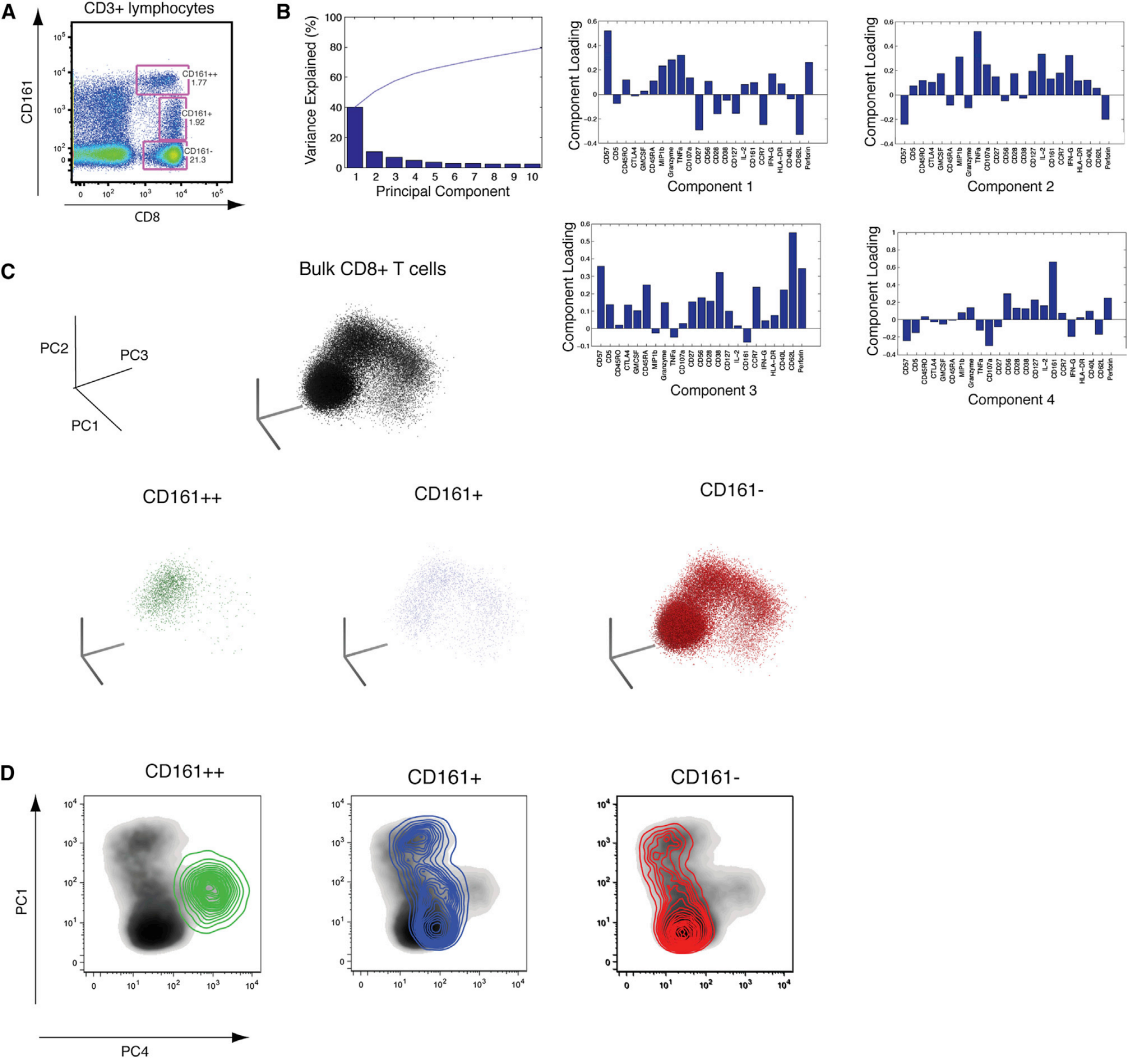
CyTOF and Principal-Component Analysis Reveals CD161++ Cells to Be Unique among CD8+ T Cells (A) Representative flow cytometry plot of the division of CD8+ T cells into three subsets based upon expression of CD161. (B) Data generated for PMA + ionomycin-stimulated CD8+ T cells were analyzed by principal-component analysis (PCA). The percent variation explained is plotted for each component (bars) and cumulatively (line), and the PCA parameter loadings (weighting coefficients) for the first four components are shown. (C) Stimulated CD8+ T cells from one representative donor are plotted on the first three components as a bulk population (black) and for the three subsets: CD161++ (green); CD161+ (blue); and CD161− (red). (D) Density plots of bulk-stimulated CD8+ T cells are plotted on component 1 and component 4 and are overlaid with contour density of plots of CD161++ (green), CD161+ (blue), and CD161− (red) cells.

Next, we wanted to investigate whether expression of CD161, and specifically different levels of expression, defined distinct CD8+ T cell phenotypes (Figure 2A). To do this, we assayed expression of 23 markers (see the Supplemental Experimental Procedures) simultaneously across phorbol 12-myristate 13-acetate (PMA) + ionomycin-stimulated CD8+ T cells by mass cytometry (CyTOF), using principal-component analysis (PCA) to integrate the patterns of expression into a smaller number of summary values (Newell et al., 2012). PCA looks for directions, or components, that cumulatively account for the variation contained within the data set, with the first four components here accounting for >60% of the total variation (Figure 2B). This analysis allowed the patterns of expression of all 23 markers to be summarized for each cell, which can then be viewed on a 2D or 3D plot, thereby allowing the different CD8+ T cell populations to be viewed in relation to one another.

When stimulated CD8+ T cells were plotted upon the first three components, CD161++ cells occupied a distinct niche compared to the other CD8+ T cell subsets (Figure 2C; Movie S1). Similarly, when plotted in 2D on component 1, which accounts for the most variation, and component 4, to which CD161 contributed the most to the variation described (component loading; Figure 2B), CD161++ cells again occupied a distinct niche among the spectrum of CD8+ T cell phenotypes (Figure 2D).

The progression in phenotypes of CD8+ T cells that expressed differing levels in CD161, and the niches they occupied, could be most clearly seen when components 1,2, and 4 were viewed together (Movie S2). In addition to CD8+ T cells that express high levels of CD161, a population of CD8+ T cells that express low, or intermediate, levels of CD161 is also apparent in the circulation (CD161+; Figure 2A). These cells displayed an overlap with the phenotypic niche of CD161++ cells (Movie S1; Figure 2D).

### The Phenotypic and Transcriptional Profile of CD161+CD8+ T Cells Overlaps with CD161++CD8+ T Cells

**Figure 3:**
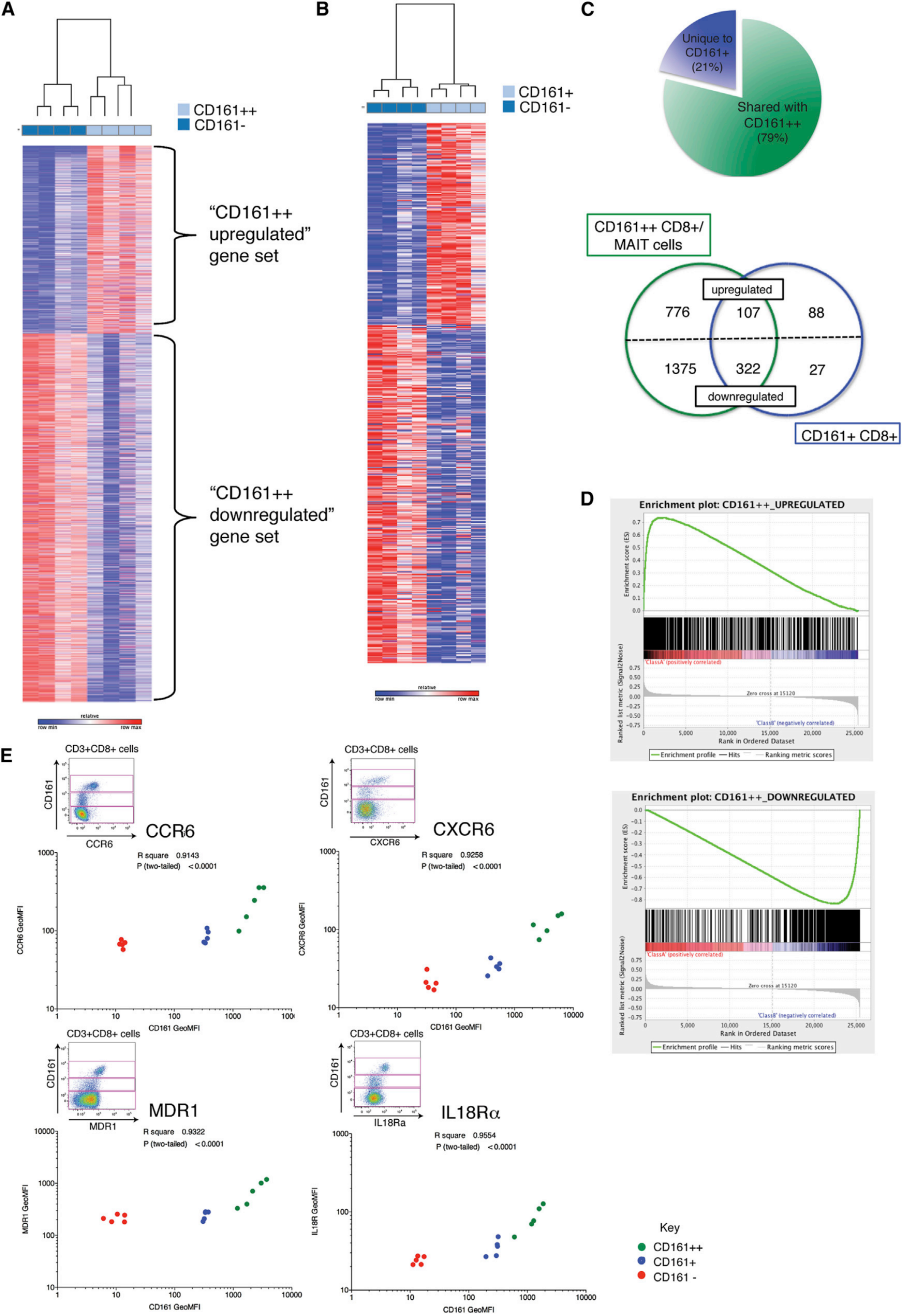
CD161+CD8+ T Cells Share a CD161++ Transcriptional Signature and Phenotypic Profile (A) Heatmap showing 3,025 significantly (p < 0.05) differentially expressed transcripts between CD161++ and CD161− CD8+ T cells in four donors. Subsets clustered by one minus Pearson correlation. (B) Heatmap showing 544 significantly differentially expressed genes between CD161+ and CD161− CD8+ T cells in the same four donors. Subsets clustered by one minus Pearson correlation. (C) Pie chart shows proportion of genes differentially expressed by CD161+ that are shared with CD161++ (79%) when compared to CD161− CD8+ T cells. Venn diagram shows breakdown of upregulated and downregulated genes unique to or shared by each subset. (D) Gene set enrichment summary plots for vsn-normalized CD161+ versus CD161− CD8+ T cell ranked genes and CD161++ upregulated (top) and downregulated (bottom) gene sets. Normalized enrichment score (NES) = 3.79, p < 0.001 upregulated genes; NES = −3.12, p < 0.001 downregulated genes. (E) GeoMFI of CD161 for CD161++ (green), CD161+ (blue), and CD161− (red) CD8+ T cell subsets correlated with GeoMFI of CCR6 (r^2^ = 0.9143; p < 0.0001), CXCR6 (r^2^ = 0.9258; p < 0.0001), MDR1 (r^2^ = 0.9322; p < 0.0001), and IL18Rα (r^2^ = 0.9554; p < 0.0001); n = 5. Representative flow cytometry plots shown for each.

To explore in more depth the phenotypic overlap of CD161+ and CD161++/MAIT CD8+ T cells, we probed genome-wide RNA expression of these CD8+ T cell populations by microarray. Previously, we identified a set of genes differentially expressed by CD161++CD8+ T cells compared to CD161− CD8+ T cells (Figure 3A; Billerbeck et al., 2010). This included the upregulated expression of RORγt, CXCR6, and IL18 receptor (IL18R). Given the dominance of MAIT cells within the CD161++CD8+ T cell population, these markers are consequently thought to be descriptive of the MAIT cell subset as well. Similarly, we performed microarray analysis on CD161+CD8+ T cells in comparison to CD161−CD8+ T cells in the same donors (Figure 3B) and identified 544 differentially expressed genes. When compared with the CD161++/MAIT cell transcriptional signature, 79% of those genes significantly differentially expressed by CD161+CD8+ T cells were shared by CD161++CD8+ T cells (Figure 3C; Tables S1 and S2). Although the majority of shared genes were downregulated, among the 107 shared upregulated genes were those characteristic of MAIT cells, including *CCR6*, *CXCR6*, *ABCB1* (encoding MDR1), and *IL18R* (Billerbeck et al., 2010, Dusseaux et al., 2011).

Next, we analyzed the gene expression data as a whole using gene set enrichment analysis (GSEA) (Subramanian et al., 2005), rather than limiting analysis to significant genes, which may miss relevant biological differences or pathway effects. GSEA orders the genes into a ranked list according to their differential expression between CD161+ and CD161− CD8+ T cells. The locations of the CD161++CD8+ T-cell-associated genes, divided into upregulated (those upregulated within CD161++CD8+ T cells) or downregulated (upregulated within CD161−CD8+ T cells; Figure 3A) genes, within this ranked list were then identified. We found significant enrichment of genes upregulated in CD161++ CD8+ T cells in CD161+CD8+ T cells (normalized enrichment score [NES] = 3.79; p < 0.001) and corresponding enrichment of downregulated genes with those genes more associated with a CD161− phenotype (NES = −3.12; p < 0.001; Figure 3D).

The correlation of CD161 expression with a number of “MAIT cell markers” identified among these shared upregulated genes was then investigated at the protein level using flow cytometric analysis. To prevent skewing by the differing proportions of each subset, defined as CD161++, CD161+, and CD161− (Figure 2A), we gated on each individual subset and plotted the expression levels of each marker, taken as geometric mean fluorescence intensity (GeoMFI), in relation to that of CD161 expression (Figure 3E). This revealed a highly significant correlation (p < 0.0001) between the levels of CD161 expressed and the expression of the CCR6 (r^2^ = 0.9143), CXCR6 (r^2^ = 0.9258), MDR1 (r^2^ = 0.9322), and IL18Rα (r^2^ = 0.9554). Therefore, expression of CD161 by CD8+ T cells correlates with a phenotypic signature that includes a distinct set of chemokine and cytokine receptors.

### Conserved Transcriptional Changes in CD161-Expressing CD4+ and TCRγδ+ T Cells

**Figure 4:**
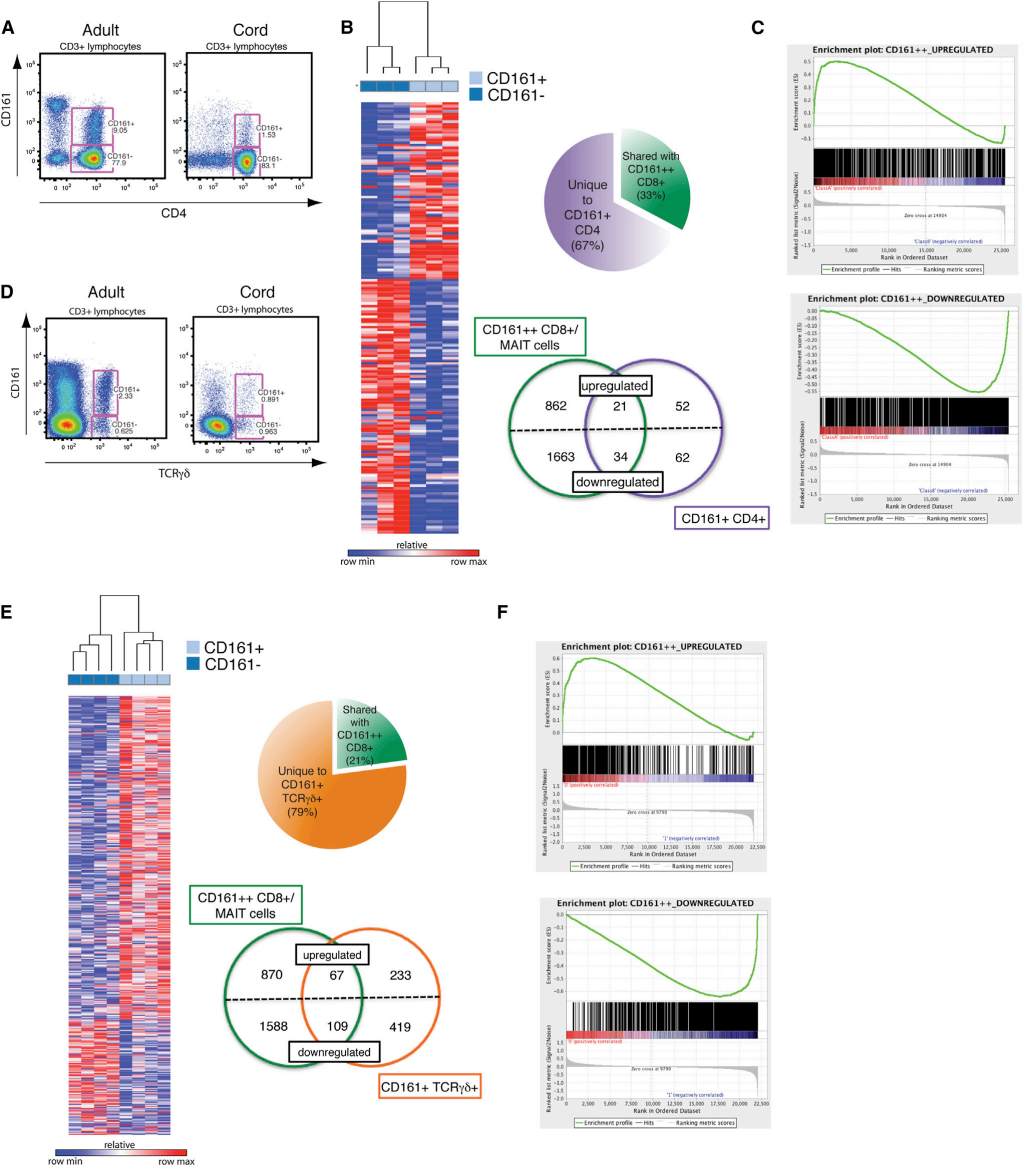
CD161+CD4+ and CD161+TCRγδ+ T Cells Are Enriched for the CD161++CD8+/MAIT Transcriptional Signature (A) Representative flow cytometry plot showing CD161+CD4+ T cells within adult and cord blood. (B) Heatmap showing 169 significantly differentially expressed genes between CD161+ and CD161− CD4+ T cells in three donors. Subsets clustered by one minus Pearson correlation. Pie chart shows proportion of genes differentially expressed by CD161+CD4+ that are shared with CD161++CD8+ (33%), when compared to their CD161− counterparts. Venn diagram shows breakdown of upregulated and downregulated genes unique to or shared by each subset. (C) Gene set enrichment summary plots for vsn-normalized CD161+ versus CD161− CD4+-T-cell-ranked genes and CD161++CD8+ upregulated (left) and downregulated (right) genes. NES = 2.45, p < 0.001 upregulated genes; NES = −3.04, p < 0.001 downregulated genes. (D) Representative flow cytometry plot showing CD161+TCRγδ+ T cells within adult and cord blood. (E) Heatmap showing 828 significantly differentially expressed genes between CD161+ and CD161− TCRγδ+ T cells in four donors. Subsets clustered by one minus Pearson correlation. Pie chart shows proportion of genes differentially expressed by CD161+TCRγδ+ that are shared with CD161++CD8+ (21%), when compared to their CD161− counterparts. Venn diagram shows breakdown of upregulated and downregulated genes unique to or shared by each subset. (F) Gene set enrichment summary plots for vsn-normalized CD161+ versus CD161− TCRγδ+-T-cell-ranked genes and CD161++CD8+ upregulated (left) and downregulated (right) genes. NES = 2.78, p < 0.001 upregulated genes; NES = −2.62, p < 0.001 downregulated genes.

A population of CD4+ T cells expressing CD161 is also apparent in the adult circulation and cord blood (Figure 4A). Both CD161+CD4+ T cells (Cosmi et al., 2008, Maggi et al., 2010) and CD161++ CD8+/MAIT cells (Billerbeck et al., 2010, Dusseaux et al., 2011) have been associated with a type-17 phenotype. Therefore, we investigated phenotypic overlap on a global scale by performing gene-expression profiling of CD161+CD4+ T cells by microarray. Genes significantly differentially expressed compared to CD161−CD4+ T cells were identified and compared to the CD161++CD8+ gene data set. CD161+CD4+ T cells shared a third of distinguishing genes with CD161++CD8+ T cells, including *IL18RAP* (Figure 4B; Tables S3 and S4). Again, GSEA demonstrated significant enrichment (p < 0.001) of those genes upregulated in CD161++CD8+ T cells in the CD161+CD4+ transcriptional profile (NES = 2.45) and downregulated genes with the CD161−CD4+ phenotype (NES = −3.04; Figure 4C), demonstrating concordance of transcriptional profiles, independently of coreceptor expression among TCRαβ+ T cells expressing CD161.

To investigate whether the CD161++/MAIT phenotype was also enriched in T cells expressing a different TCR, we extended our analysis to TCRγδ+ T cells. Again, both CD161+ and CD161− TCRγδ+ T cells are evident within the adult circulation and cord blood (Figure 4D). Gene-expression analysis was performed on sorted CD161+ and CD161− TCRγδ+ T cells and those genes significantly differentially expressed identified (Figure 4E). Approximately a fifth of these were again shared with the CD161++CD8+ gene data set, including *IL18R1* and *ABCB1*, which encodes MDR1 (Tables S5 and S6). As seen for CD8+ and CD4+ T cells, GSEA revealed the CD161++CD8+ gene set to be significantly enriched (p < 0.001) in CD161+TCRγδ+ T cells, corresponding to both upregulated (NES = 2.78) and downregulated (NES = −2.62) genes (Figure 4F), despite a reduced number of genes being shared.

Overall, CD161 expression can distinguish distinct populations of T cells within each T cell lineage, whether CD8+, CD4+, or TCRγδ+, with a defined CD161++/MAIT-cell-associated transcriptional signature enriched within this CD161-positive subset. Moreover, comparison of the leading edge gene set (the core set of genes that account for this enrichment) from each T cell population distinguished a core of 124 upregulated (Table 1) and 199 downregulated (Table S7) genes commonly enriched in all CD161-expressing T cells and which therefore defines the CD161-associated transcriptional signature. This included upregulated expression of *ABCB1* (MDR1), *RORC* (RORγ), *ZBTB16* (PLZF), *IL12RB2*, and *IL18R1*.

**Table 1:** Core Transcriptional Signature of CD161-Associated Upregulated Genes

*ABCA2*	*CTNNA1*	*IGFBP4*	*MYO1F*	*SESN1*
*ABCB1*	*CTSH*	*IL12RB2*	*NADK*	*SIPA1L2*
*ACTN4*	*CXCR6*	*IL15*	*NBEAL2*	*SLAMF1*
*ADCY9*	*CXXC5*	*IL18R1*	*NETO2*	*SLC1A5*
*ADRB2*	*CYB561*	*IL18RAP*	*NPC1*	*SLC2A8*
*AGPAT4*	*CYFIP1*	*IL23R*	*PBX4*	*SLC7A5*
*ALCAM*	*DENND3*	*IRAK2*	*PERP*	*SLCO3A1*
*ARNTL*	*DUSP5*	*JSRP1*	*PHACTR2*	*SMAD3*
*AUTS2*	*EIF2C4*	*KIAA1539*	*PHLDA1*	*SMAD7*
*B4GALT1*	*ELOVL4*	*KIF5C*	*PIK3AP1*	*SNAI3*
*CACNA2D2*	*ELOVL6*	*KLRB1*	*PLCB1*	*SYNE2*
*CACNA2D4*	*ERN1*	*KLRG1*	*PLEKHG3*	*SYTL2*
*CAMTA1*	*FAM43A*	*LAG3*	*PLXNC1*	*THBS1*
*CAPN12*	*FAS*	*LATS2*	*PODXL*	*TLE1*
*CCR2*	*FOSL2*	*LONRF3*	*PRDM1*	*TNF*
*CD58*	*GALM*	*LTB4R*	*PRDM8*	*TNFRSF18*
*CD72*	*GBP5*	*LTK*	*PRF1*	*TNFSF14*
*CERK*	*GFPT2*	*MAFF*	*PTGDS*	*TPM2*
*CFH*	*GPR65*	*MAP3K5*	*PTPRM*	*VCL*
*CLCF1*	*GPR68*	*MAP3K8*	*RAB11FIP1*	*VLDLR*
*CLIC5*	*GTF3C1*	*MATK*	*RASD1*	*WNT1*
*COL5A1*	*GZMA*	*ME1*	*RHBDF2*	*YPEL1*
*COL5A3*	*GZMK*	*METRNL*	*RORA*	*ZBTB16*
*COLQ*	*IFI44*	*MICALCL*	*RORC*	*ZDHHC14*
*CREB3L2*	*IFNGR1*	*MYO1D*	*RPPH1*	

Leading-edge analysis was performed on the enriched CD161++CD8+ T cell upregulated gene set in all T cell subsets. The leading-edge gene set was compared from each T cell population and a core set of 124 genes identified and listed in alphabetical order. Those referred to in the text are underlined.

### A Common Functional Correlate of CD161 Expression in All T Cell Subsets

**Figure 5:**
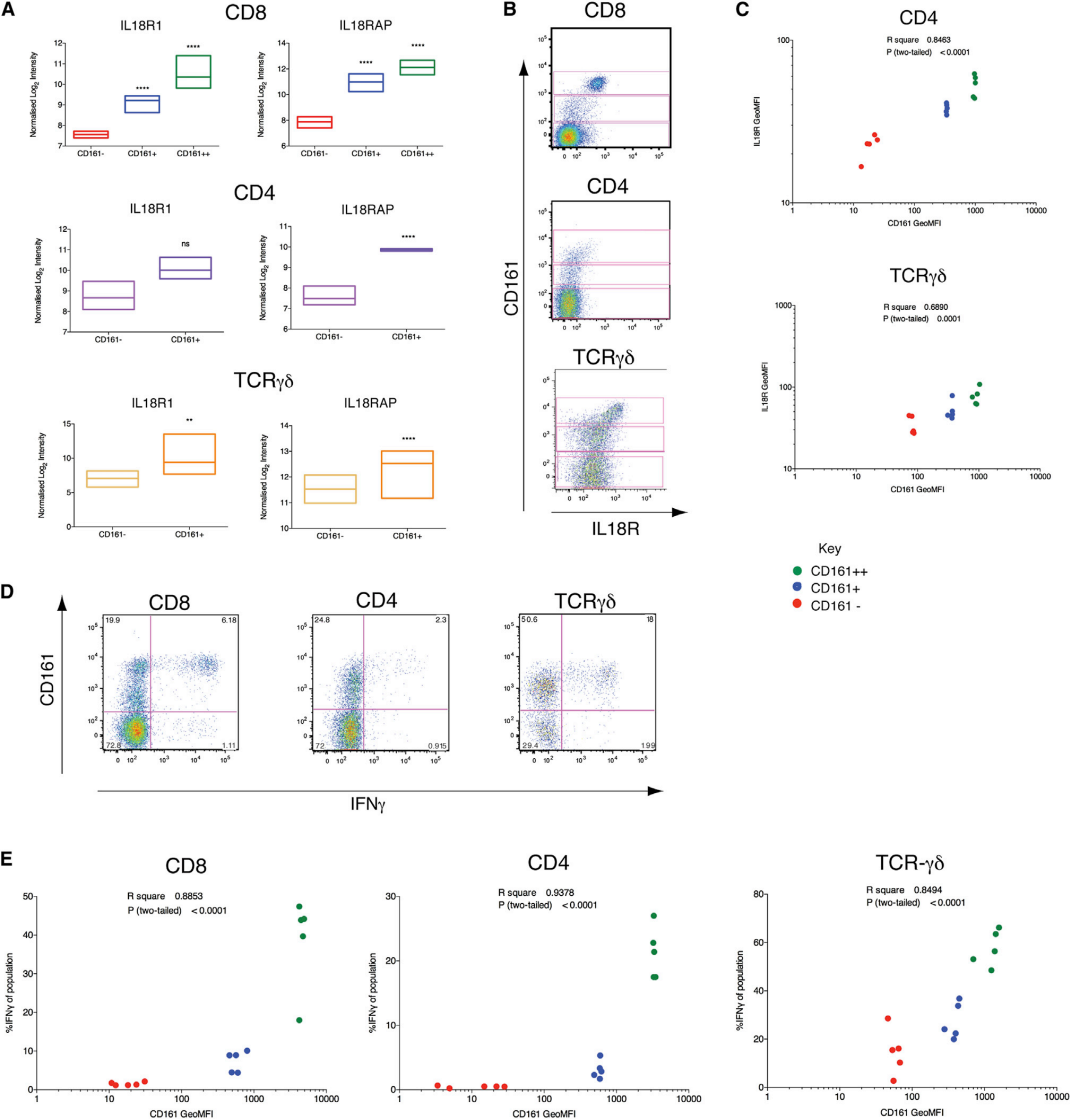
CD161-Positive T Cell Subsets Express Elevated Levels of IL18Rα and Respond to Stimulation by IL-12+IL-18 (A) Relative expression levels of IL18R subunits by CD161-positive and CD161-negative sorted T cells and statistical significance from mRNA expression analyses after normalization and correction for multiple testing. ^∗∗^p < 0.01; ^∗∗∗∗^p < 0.0001. Floating bars show minimum and maximum values, with a line at the mean. (B) Representative flow cytometry plots for IL18Rα expression in each T cell lineage, revealing CD161++, CD161+, and CD161− subsets in each (gated). (C) GeoMFI of CD161 for CD161++ (green), CD161+ (blue), and CD161− (red) subsets in CD4+ and TCRγδ+ T cells correlated with GeoMFI of IL18Rα. CD4 r^2^ = 0.8463, p < 0.0001; TCRγδ r^2^ = 0.6890, p < 0.0001 (n = 5). (D) PBMCs were stimulated overnight with 50 ng/ml IL-12+IL-18. Representative flow cytometry plots for IFNγ expression by gated CD8+, CD4+, and TCRγδ+ CD3+ live lymphocytes. (E) GeoMFI of CD161 for CD161++ (green), CD161+ (blue), and CD161− (red) subsets in CD8+, CD4+, and TCRγδ+ T cells correlated with percentage of each subset expressing IFNγ in response to overnight stimulation with IL-12+IL-18. CD8 r^2^ = 0.8853, p < 0.0001; CD4 r^2^ = 0.9378, p < 0.0001; TCRγδ r^2^ = 0.8494, p < 0.0001 (n = 5). See also Figure S2.

IL18R was one of the defining components of the leading edge gene set associated with CD161 expression. The receptor is composed of two subunits: IL18Rα and IL18RAP. *IL18R1* (IL18Rα) was significantly upregulated in CD161+TCRγδ+ (p < 0.01) and both CD161-positive populations of CD8+ (p < 0.0001) T cells and *IL18RAP* significantly upregulated in all CD161-expressing T cell subsets (p < 0.0001; Figure 5A). Expression was confirmed at the protein level by flow cytometry, which also revealed a previously unappreciated CD161++IL18Rα++ subset among CD4+ and TCRγδ+ T cells (Figure 5B). Gating of these three subsets (CD161++, CD161+, and CD161−) revealed a significant correlation between CD161 expression and IL18Rα expression (Figure 5C) among CD4+ (p < 0.0001) and TCRγδ+ (p = 0.0001) T cells, as for CD8+ T cells (Figure 3E).

We recently described that elevated expression of IL18R enables CD161++CD8+/MAIT cells to be activated by IL-12+IL-18 in a TCR-independent manner (Ussher et al., 2014). As genes encoding both subunits of the IL-18R and *IL12RB2* were contained within the core leading edge set of CD161-associated upregulated genes (Table 1), we assayed IFNγ production in response to overnight stimulation with IL-12+IL-18 in CD8+, CD4+, and TCRγδ+ T cells. IFNγ production was apparent by intracellular cytokine staining in all three T cell lineages and particularly apparent within the CD161++ population of each (Figure 5D). Although CD4+ MAIT cells do exist (Reantragoon et al., 2013), IFNγ responses were not restricted to Vα7.2+CD4+ T cells (Figure S2). We correlated this response to IL12+IL18 with the levels of CD161 expressed by gating on the three CD161 populations (CD161++, CD161+, and CD161−) within each T cell lineage, as previously (Figure 5B). As expected, there was a significant correlation between levels of CD161 expressed by each population and the percentage of that population expressing IFNγ in response to IL-12+IL-18 (Figure 5E), with the greatest responses seen by the CD161++ population in each.

Together, these results demonstrate CD161-positive T lymphocytes to be related both in terms of gene expression and function, including elevated expression of IL18R in the resting state. This is linked to their shared ability to make an innate response to cytokine stimulation by IL-12+IL-18, which is independent of the TCR yet correlated to expression of CD161.

### Regulation by CD161

**Figure 6:**
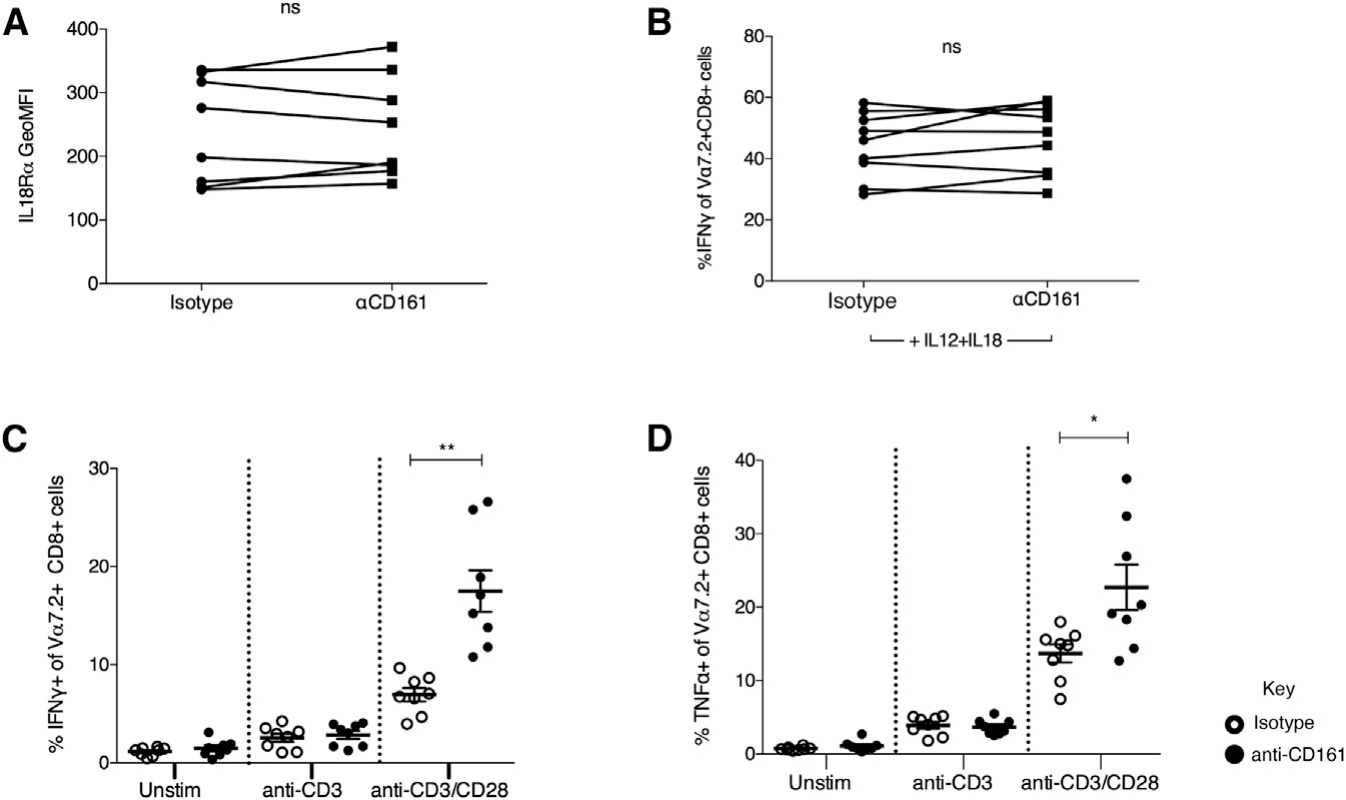
CD161 Regulates TCR-Dependent, but Not TCR-Independent, Responses (A) GeoMFI of IL18Rα on Vα7.2+CD8+CD3+ cells incubated for 18 hr with biotin beads coated with either IgG2a isotype or anti-CD161. ns, not significant by paired t test (n = 9). (B) Percentage of Vα7.2+CD8+CD3+ cells expressing IFNγ when cultured with 50 ng/ml IL-12+IL-18 for 18 hr in the presence of biotin beads coated with either IgG2a isotype or anti-CD161. ns, not significant by paired t test (n = 9). (C and D) Percentage of Vα7.2+CD8+CD3+ cells expressing IFNγ (C) or TNF-α (D) when cultured overnight with TCR-stimulating biotin beads, coated either with anti-CD3 or anti-CD3 + anti-CD28, in combination with IgG2a isotype (open circles) or anti-CD161 (filled circles). ^∗^p < 0.05 and ^∗∗^p < 0.01 by repeated-measures one-way ANOVA (n = 8). Data are represented as mean ± SEM. See also Figure S3.

Expression of CD161 by NK cells is generally accepted to negatively regulate NK cell functions (Aldemir et al., 2005, Lanier et al., 1994, Rosen et al., 2005), whereas the effect of CD161 ligation on T cell function is less clear (Aldemir et al., 2005, Exley et al., 1998, Le Bourhis et al., 2013, Rosen et al., 2008). To investigate the role of CD161 in the response to IL12+IL18, we ligated CD161 by adding biotin beads coated with anti-CD161 into the culture. Ligation of CD161, either by its ligand LLT1 or anti-CD161, induces transient downregulation in CD161 expression (Figure S3). Therefore, this analysis could only be reliably performed on MAIT cells, which can be tracked independently through expression of Vα7.2. Ligation of CD161 had no effect on MAIT cell expression of IL18R (Figure 6A) or IFNγ production in response to IL-12+IL-18 (Figure 6B). Previously, CD161 has been shown to regulate responses only in the context of TCR stimulation (Aldemir et al., 2005, Exley et al., 1998); therefore, we examined the effect of CD161 ligation in addition to stimulation through the TCR. Ligation of CD161 induced a significant increase in IFNγ (p < 0.01) and tumor necrosis factor alpha (TNF-α) (p < 0.05) production when MAIT cells were stimulated with anti-CD3 and anti-CD28 compared to cells stimulated with anti-CD3 and anti-CD28 alone (Figures 6C and 6D). This suggests, along with published data (Aldemir et al., 2005, Exley et al., 1998) and other data using soluble anti-CD161 (data not shown), that CD161 can act as a costimulatory receptor to increase the response to TCR stimulation.

## Discussion

Conventionally, T lymphocyte subsets have been identified and divided based upon the expression of sets of proteins, including cell surface markers, cytokines, and nuclear transcription factors. The C-type lectin CD161 is expressed by a large proportion of cells within each of these previously defined subsets, including MAIT, CD4+ and CD8+, and both TCRαβ+ and TCRγδ+ T cells. Despite the diverse phenotypes and functions of these individual T cell subsets, associated with their recognition of distinct antigens on specific antigen-presenting molecules, we have demonstrated these cell lineages to be related by a shared transcriptional signature and innate-like function and marked by expression of CD161.

Among those T cells expressing the highest levels of CD161 is the population of innate-like, unconventional T cells known as MAIT cells. This population is distinct in terms of TCR expression (Tilloy et al., 1999), antibacterial function (Le Bourhis et al., 2010), and type-17-related transcriptional and phenotypic profile (Billerbeck et al., 2010). This study represents a phenotypic description of CD161++CD8+ MAIT cells by multiparametric mass cytometry (CyTOF). Despite inclusion of only “conventional” T cell markers within the 23-parameter panel, rather than the unique markers characteristic of MAIT cells, PCA emphasized the distinctiveness of this population, which segregates within a discrete niche in the spectrum of CD8+ T cell phenotypes. It would be of interest to identify the location of the Vα7.2− CD161++CD8+ T cell population in relation to Vα7.2+ MAIT cells within the spectrum of CD8+ T cell phenotypes, which was precluded here by the absence of an isotope-tagged Vα7.2 antibody.

Although the Vα7.2− CD161++CD8+ T cell population is highly heterogeneous in terms of both TCR α and β chain expression, both Vα7.2+ and Vα7.2− CD161++ subsets expressed a memory phenotype and elevated levels of the polarizing transcription factors RORγt and PLZF. The central role of PLZF in directing the innate-like functions of NKT and MAIT cells (Savage et al., 2008) and other innate lymphocytes (Constantinides et al., 2014) has recently been revealed. The shared expression by Vα7.2− CD161++CD8+ T cells, early in development (Walker et al., 2012), suggests that these cells may also display innate characteristics. We investigated whether diverse Vα7.2− cells represented a population analogous to type II NKT cells (Godfrey et al., 2004), which recognize identical ligands to invariant NKT cells, an additional innate-like lymphocyte subset, despite diverse TCR expression (Gadola et al., 2002). It appears that Vα7.2− CD161++CD8+ T cells are not MR1 responsive in the majority of donors; however, this does not rule out the possibility that this population may be reactive to other nonclassical ligands and warrants further investigation. Nonetheless, like Vα7.2+ MAIT cells, Vα7.2− CD161++CD8+ T cells are specifically activated by stimulation by IL-12+IL18, as shown here and elsewhere (Ussher et al., 2014), representing a shared innate-like feature of both populations of CD161++CD8+ T cells.

As MAIT cells displayed a phenotypic and functional relationship to Vα7.2− CD161++CD8+ T cells, we utilized mRNA microarray analysis to examine the relationship of MAIT cells to other CD161-expressing T cell subsets. Previously, whole transcriptome analysis by microarray has revealed that memory T and B cells display a transcriptional signature that is shared with hematopoietic stem cells (Luckey et al., 2006), and transcripts that distinguish T and B cells are also expressed among other lymphocyte cell types (Painter et al., 2011). This indicates that expression patterns and their consequent phenotypes may be shared across previously defined divisions and may further be suggestive of shared functional characteristics. In a similar way, analysis of mRNA microarray data for CD161-positive T cells, including TCRγδ+, CD4+, and both populations of CD8+ T cells, revealed a CD161-associated transcriptional signature enriched in all T lymphocytes expressing CD161 and led to the identification of a shared functional attribute.

The core transcriptional profile of CD161-expressing T cells included genes for subunits of both the IL-12 and IL-18 receptors, with high IL18Rα expression conserved across CD161-positive T cells of all lineages. Expression of this receptor was related to the ability of these cells to respond to IL-18 in combination with IL-12, inducing the innate TCR-independent production of IFNγ. Although this has previously been described for CD161++ CD8+ T cells, including MAIT cells (Ussher et al., 2014) and confirmed here for Vα7.2−CD161++, this has not been described for other T cell subsets, with CD161++ subsets in all lineages, identified among CD4+ and TCRγδ+ T cells, displaying the highest response levels. This establishes CD161 as a marker of cells with an enhanced “innate” ability to respond to this stimulus. Furthermore, this TCR-independent pathway suggests a means by which these populations may be activated in the inflammatory conditions in which they have been implicated (Annibali et al., 2011, Billerbeck et al., 2010, Kang et al., 2012, Kleinschek et al., 2009, Poggi et al., 1999).

The significance of CD161 expression by T cells has not yet been fully determined. However, CD161 has previously been applied as a marker of IL-17-expressing T cells (Maggi et al., 2010), particularly Th17 cells (Cosmi et al., 2008), and may be involved in the induction of this phenotype (Bai et al., 2014). Whereas CD161 was shown here to mark cells with an innate-like ability to respond to cytokine stimulation, ligation of CD161 had no effect on this response. Instead, CD161 was demonstrated to function as a costimulatory receptor in the context of TCR stimulation. This effect may differ from those shown by others (Le Bourhis et al., 2013, Rosen et al., 2008) due to differences in the presentation of anti-CD161 or in the clones used (see Figure S3D).

MAIT cells have previously been described to be hyporesponsive to stimulation through the TCR (Turtle et al., 2011), a feature recently suggested to characterize all innate-like lymphocytes (Wencker et al., 2014). This was related to low expression levels of genes for multiple proteins that positively regulate TCR signaling, including the genes *ITK* and *MAL* (Turtle et al., 2011). Interestingly, these genes were also contained within the set of core downregulated genes we identified here, being shared between all CD161-expressing T cells (Table S7). If similarly TCR hyporesponsive, then by acting as a costimulatory receptor, CD161 may help to overcome the reduced reactivity of the TCR in these innate-like T cells (Wencker et al., 2014). Induction of TCR hyporesponsiveness during development of innate-like T cells has also been associated with the ability to respond to cytokine stimulation and interestingly enabled identification by Wencker et al. (2014) of an innate-like murine TCRγδ+ T cell subset that also responded selectively to IL-12 and IL-18.

In contrast to the current paradigms of T cell subdivision, this study identifies a phenotype and function that is shared across T cell lineages and marked by expression of CD161. Here, we describe CD161 to identify cells with a shared transcriptional profile, including high expression of IL18R, and capable of making innate-type responses to cytokine stimulation. Therefore, expression of CD161 marks human T cells with a distinct phenotype that is independent of lineage and identifies a family of related lymphocytes with innate characteristics that includes MAIT cells.

## Experimental Procedures

### Cells

PBMCs were obtained from adults (whole blood leukocyte cones; NHS Blood and Transplant), 24 month olds (prospective birth cohort; Saghafian-Hedengren et al., 2010), and umbilical cord blood samples (Stem Cell Services, NHS Blood and Transplant) after appropriate ethical review. These were rested overnight or stored in liquid nitrogen until required.

THP-1 cells (ECACC) were incubated with paraformaldehyde-fixed *E. coli* (DH5α; Invitrogen) at 25 bacteria per cell overnight. PMBCs (24-month donors) or sort-purified CD8+ T cells (adults; sorted on a MoFlo; Beckman Coulter Genomics) were then added for a 5 hr coculture. Anti-MR1 (clone 26.5, kindly provided by T.H. Hansen) or IgG2a isotype control (BD Biosciences) was added at 10 μg/ml. For IL-12 + IL-18 stimulations, IL-12 (Miltenyi Biotec) and IL-18 (R&D Systems) were added at 50 ng/ml overnight. In all cases, brefeldin A (eBioscience) was added for the final 4 hr of incubation.

### TCR Sequencing

T cell receptor sequencing of single cells was performed as previously described (Wang et al., 2012). In brief, Vα7.2+ and Vα7.2− CD161++CD8+ T cells were sorted (MoFlo; Beckman Coulter) as single cells into wells of a 96-well plate containing 2.5 μl of iScript cDNA Synthesis reaction mixture (Bio-Rad) with 0.1% Triton X-100. TCR transcripts for each cell were amplified by multiplex nested PCR using one unit of Platinum Taq DNA polymerase High Fidelity, 10× PCR buffer, 2 mM magnesium sulfate and 0.2 mM deoxynucleotide triphosphate (Invitrogen), 2.5 pmol of each external TRAV and TRBV primer and 10 pmol of each external TRAC and TRBC in the first round, or the same concentration of either internal TRAV and TRAC or internal TRBV and TRBC primers in the second round (primers as detailed in Wang et al., 2012). The PCR conditions were 95°C for 2 min, followed by 35 cycles of 95°C for 20 s, 52°C for 20 s, 72°C for 45 s, followed by one cycle of 72°C for 7 min. PCR products were purified and sequenced with the corresponding TRAC or TRBC primer.

### Microarrays

Cell sorting, RNA extraction, and microarray analysis of CD161 subsets within CD8+ T cells was performed as previously described (Billerbeck et al., 2010).

CD4+ T cells were isolated from three donors by magnetic bead enrichment (EasySep; STEMCELL Technologies) and CD161 subsets selected by staining anti-CD161-phycoerythrin (PE); Beckman Coulter) followed by purification with anti-PE MicroBeads (Miltenyi Biotec). Purity, as determined by flow cytometry, was >95% for all samples. Total RNA was extracted using an RNeasy Mini Kit (QIAGEN) and quality measured using the ND 1000 Spectrophotometer (Saveen Werner). cRNA was amplified and purified using Illumina TotalPrep RNA amplification kit (Ambion) and hybridized to the Illumina HumanWG-6 v3.0 Expression BeadChip (Illumina).

For gene-expression profiling of TCRγδ+ T cell subsets, T cells were enriched from PBMCs from four donors as per the manufacturer’s instructions (EasySep T cell enrichment kit; STEMCELL Technologies). CD3+TCRγδ+CD161+ or CD161− were sorted using a FACSAria (BD Biosciences). Cell pellets were snap frozen and sent to Miltenyi Biotec Genomic Services (Bergisch Gladbach) for RNA extraction and hybridization to Agilent Whole Human Genome Oligo Microarray.

Microarray features were normalized using variance-stabilizing normalization (vsn) (Huber et al., 2002) using R (http://cran.r-project.org/) and Bioconductor (http://bioconductor.org) packages. Gene lists of interest were generated from those genes >2-fold up/downregulated and with a p < 0.05 for comparison using the Venn diagram module of GenePattern (Gould et al., 2006). Heatmaps were generated using GENE-E (http://www.broadinstitute.org/cancer/software/GENE-E/index.html). Gene set enrichment analysis was performed using GSEA version 2.0.14 (Subramanian et al., 2005).

### CyTOF

PBMCs were stimulated for 3 hr with 150 ng/ml PMA + 1 μM ionomycin in RPMI plus brefeldin A and monensin (eBioscience), 2.5 μg/ml anti-CD107α, 1.25 μg/ml anti-CD107b (BD Bioscience), and 10 μM TAPI-2 (VWR International). Following stimulation, cells were resuspended in cytometry buffer (PBS + 0.05% sodium azide + 2 mM EDTA + 2% fetal calf serum) and stained with isotope-tagged antibodies before being acquired on the CyTOF. For the detailed protocol, see Newell et al. (2012) and the Supplemental Experimental Procedures.

### Statistical Analysis

Statistical analysis was performed using Prism version 6 software (GraphPad). Data are represented as mean ± SEM. ^∗∗∗∗^p < 0.0001; ^∗∗∗^p < 0.001; ^∗∗^p < 0.01; ^∗^p < 0.05; and ns, not significant, as stated in figure legends.

## Author Contributions

J.R.F. designed, performed, and analyzed experiments and wrote the manuscript. K.E.S. designed, performed, and analyzed the CD161 ligation experiments. V.M.F., N.R., and Y.-H.K. performed the microarrays. All remaining authors contributed to specific experiments. P.K. designed experiments and provided overall guidance.

## Accession Numbers

The NCBI Gene Expression Omnibus accession number for the microarray data reported in this paper is GSE62099.

## Supplemental Information

Document S1. Supplemental Experimental Procedures, Figures S1–S3, and Table S7

Table S1. Transcriptional Comparison of CD161+ versus CD161− CD8+ T Cells, Upregulated Genes Shared or Unique from CD161++CD8+ T Cells, Related to Figure 3

Table S2. Transcriptional Comparison of CD161+ versus CD161− CD8+ T Cells, Downregulated Genes Shared or Unique from CD161++CD8+ T Cells, Related to Figure 3

Table S3. Transcriptional Comparison of CD161+ versus CD161− CD4+ T Cells, Upregulated Genes Shared or Unique from CD161++CD8+ T Cells, Related to Figure 4

Table S4. Transcriptional Comparison of CD161+ versus CD161− CD4+ T Cells, Downregulated Genes Shared or Unique from CD161++CD8+ T Cells, Related to Figure 4

Table S5. Transcriptional Comparison of CD161+ versus CD161− TCRγδ+ T Cells, Upregulated Genes Shared or Unique from CD161++CD8+ T Cells, Related to Figure 4

Table S6. Transcriptional Comparison of CD161+ versus CD161− TCRγδ+ T Cells, Downregulated Genes Shared or Unique from CD161++CD8+ T Cells, Related to Figure 4

Movie S1. 3D-PCA PyMOL Dot Plot 1, Related to Figure 2 Each dot represents a single cell plotted in 3D according to the first three principal components; cells are colored according to gating of the three defined populations of CD8+ T cells: CD161++ (green); CD161+ (blue); and CD161− (red).

Movie S2. 3D-PCA PyMOL Dot Plot 2, Related to Figure 2 Each dot represents a single cell plotted in 3D according to the principal components 1, 2, and 4; cells are colored according to gating of the three defined populations of CD8+ T cells: CD161++ (green); CD161+ (blue); and CD161− (red).

Document S2. Article plus Supplemental Information
