# Supplementary material for: CD161 Defines a Transcriptional and Functional Phenotype across Distinct Human T Cell Lineages
Source: Cell Rep. 2014 Oct 23;9(3):1075–88. doi: 10.1016/j.celrep.2014.09.045 (PMC4250839; doi:10.1016/j.celrep.2014.09.045)
Supplement: Document S1. Supplemental Experimental Procedures, Figures S1–S3, and Table S7 [file mmc1.pdf]

Cell Reports, Volume *9*

Supplemental Information

CD161 Defines a Transcriptional and Functional Phenotype across Distinct Human T Cell Lineages

Joannah R. Fergusson, Kira E. Smith, Vicki M. Fleming, Neil Rajoriya, Evan W. Newell, Ruth Simmons, Emanuele Marchi, Sophia Björkander, Yu-Hoi Kang, Leo Swadling, Ayako Kurioka, Natasha Sahgal, Helen Lockstone, Dilair Baban, Gordon J. Freeman, Eva Sverremark-Ekström, Mark M. Davis, Miles P. Davenport, Vanessa Venturi, James E. Ussher, Christian B. Willberg, and Paul Klenerman

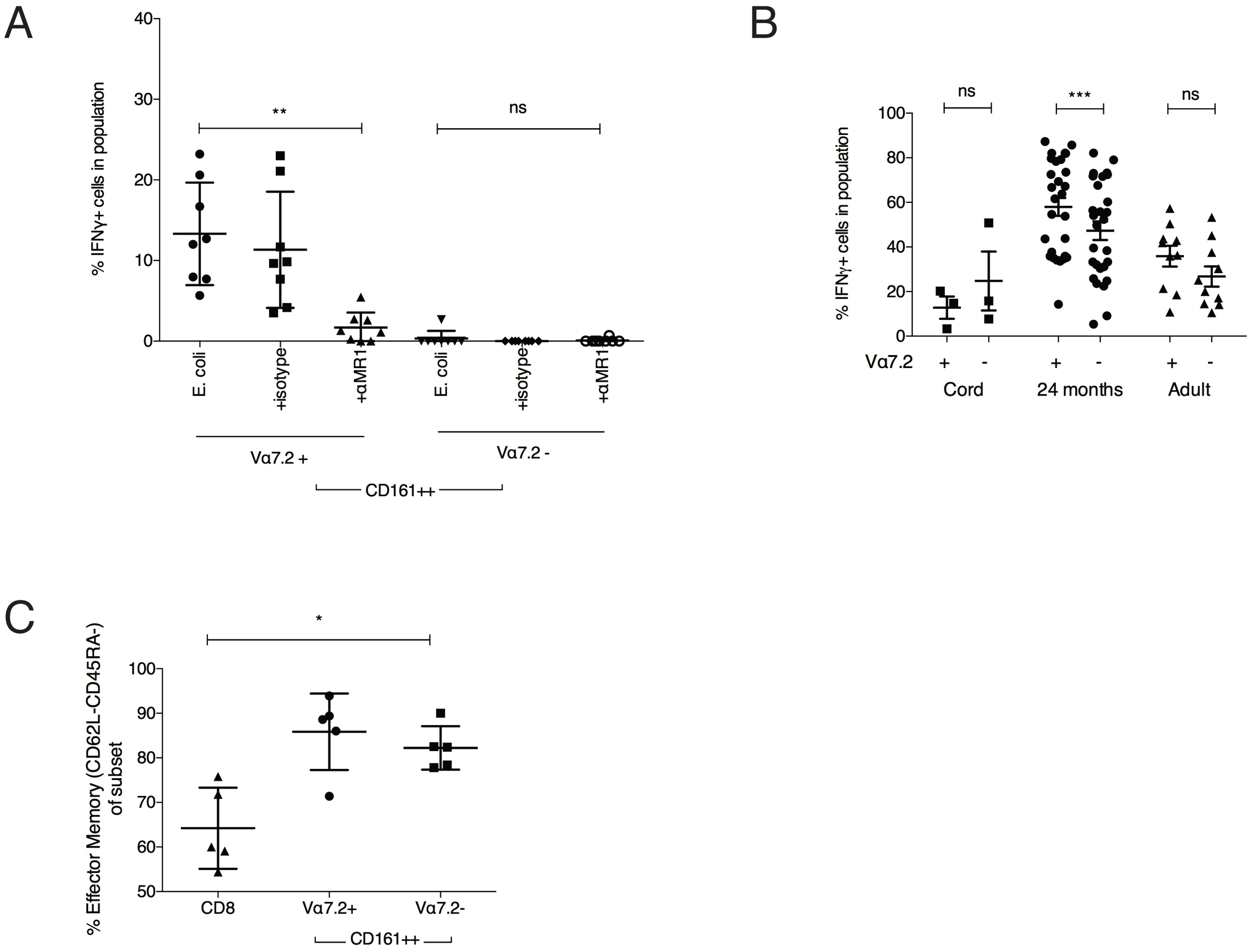

Figure S1, related to Figure 1: Vα7.2– CD161++CD8+ T cell phenotype. A) PBMCs from 24 month old donors were co-cultured with THP1s exposed to *E. coli* for 5hr in the presence or absence of anti-MR1 antibody or isotype control (10µg/ml). Data shows percentage IFNγ expression from gated subsets. (n=8) B) Percentage IFNγ expression of PBMCs cultured overnight with IL-12 + IL-18 (50ng/ml) from cord (n=3), 24 month (n=28) and adult (n=10) blood. ns=non-significant, ***p<0.001 by paired t-tests. C) Percentage of CD8+ T cell subsets displaying an effector memory (CD62L– CD45RA–) phenotype (n=5) *p<0.05, ** p<0.01 by one-way ANOVA with Dunnett's multiple comparisons test.

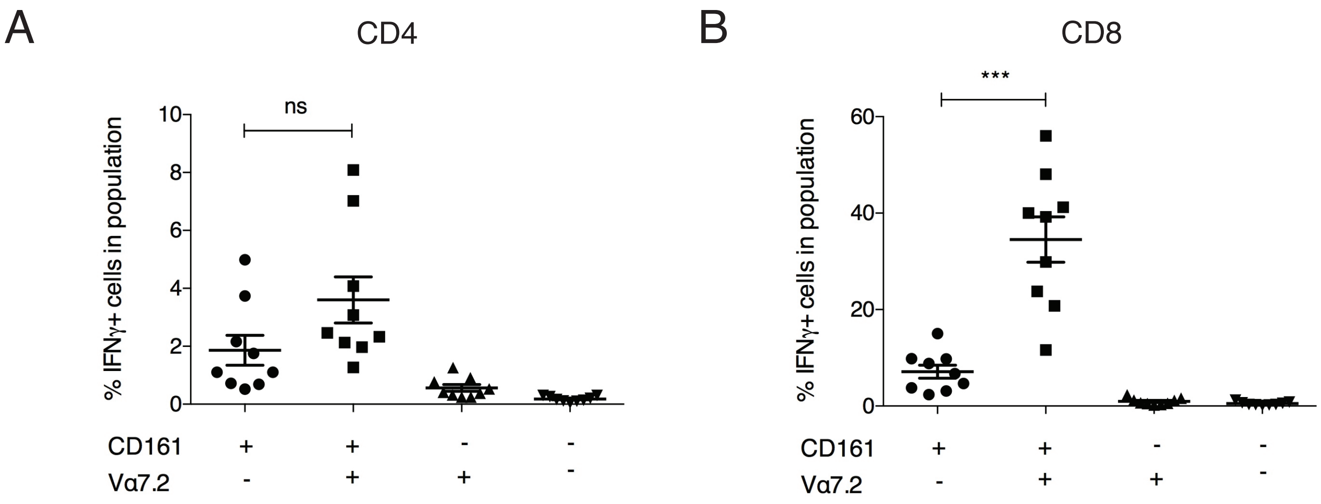

Figure S2, related to Figure 5: IL-12+IL-18 induces responses from Vα7.2+ and Vα7.2- CD161+ T cells. PBMCs were incubated overnight with 50ng/ml of IL-12 + IL-18 and IFNγ production assayed by intracellular cytokine staining. Percentage IFNγ-producing cells of CD161+/CD161− and either Vα7.2+/Vα7.2− populations was calculated and plotted for CD4+ (A) and CD8+ (B) T cells. *** $p<0.001$, ns = not significant with Tukey's multiple comparisons test (n=9).

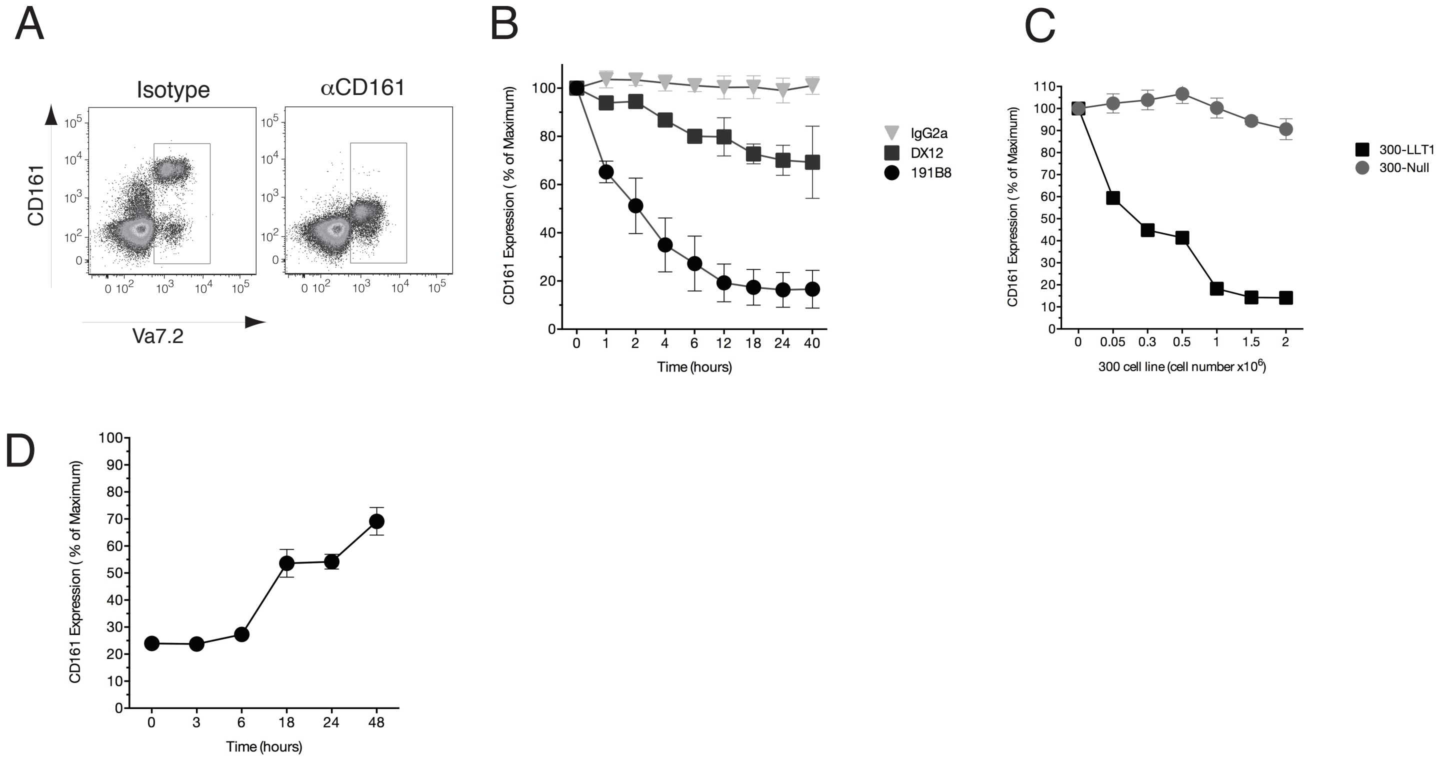

Figure S3, related to Figure 6: Transient downregulation of CD161 upon ligation. A) Representative flow cytometry plot showing downregulation of CD161 upon ligation by biotin beads coated with anti-CD161 (clone: 191B8) but not with IgG2a isotype. B) Percentage of maximum CD161 expression after incubation with either IgG2a isotype, or anti-CD161 clone DX12 or 191B8, which induces rapid down-regulation of CD161. C) Percentage of maximum CD161 expression upon incubation with 300 cell line expressing the CD161 ligand, LLT1, or the parental cell line (300-Null) only. D) After a 3hr co-incubation, the ligand was removed by enriching for Vα7.2+ cells. These cells were incubated for 0-48hr before staining for CD161. Expression levels from those cells incubated with 300-LLT1 were compared to those incubated with 300-Null, demonstrating recovery of CD161 expression levels over time.

Table S1, related to Figure 3: Transcriptional comparison of CD161+ vs CD161− CD8+ T cells, upregulated genes shared or unique from CD161++CD8+ T cells.

Table S2, related to Figure 3: Transcriptional comparison of CD161+ vs CD161− CD8+ T cells, downregulated genes shared or unique from CD161++CD8+ T cells.

Table S3, related to Figure 4: Transcriptional comparison of CD161+ vs CD161− CD4+ T cells, upregulated genes shared or unique from CD161++CD8+ T cells.

Table S4, related to Figure 4: Transcriptional comparison of CD161+ vs CD161− CD4+ T cells, downregulated genes shared or unique from CD161++CD8+ T cells.

Table S5, related to Figure 4: Transcriptional comparison of CD161+ vs CD161− TCRγδ+ T cells, upregulated genes shared or unique from CD161++CD8+ T cells.

Table S6, related to Figure 4: Transcriptional comparison of CD161+ vs CD161− TCRγδ+ T cells, downregulated genes shared or unique from CD161++CD8+ T cells.

Movie S1, related to Figure 2: 3D-PCA PyMOL Dot plot 1. Each dot represents a single cell plotted in 3D according to the first three principal components, cells are coloured according to gating of the three defined populations of CD8+ T cells: CD161++ (green), CD161+ (blue) and CD161− (red).

Movie S2, related to Figure 2: 3D-PCA PyMOL Dot plot 2. Each dot represents a single cell plotted in 3D according to the principal components 1, 2 and 4, cells are coloured according to gating of the three defined populations of CD8+ T cells: CD161++ (green), CD161+ (blue) and CD161− (red).

ABCD2	*COQ6*	*LEPROTL1*	*PKIA*	*SNPH*
ACCN2	*CRLF3*	*LIMS2*	*PLAG1*	*SNRPN*
ACSM3	*CYP2J2*	*LOC338799*	*PLEKHB1*	*SOX8*
ACTN1	*DSC1*	*LOC388588*	*PPAP2A*	*SPINK2*
AEBP1	*EFHD1*	*LOC401431*	*PPAPDC2*	*SPINT2*
AGMAT	*EPHX2*	*LOC641518*	*PPP1R3E*	*SRPK2*
ALKBH7	*ERLIN2*	*LRRN3*	*PRIM1*	*STAT1*
ANAPC13	*ESD*	*LYPD3*	*PRKACB*	*SUCLG2*
APBA2	*ETS1*	*MAD1L1*	*PRKAR1B*	*SULT1B1*
APEX1	*FAM55C*	*MAD2L1*	*PRKCA*	*SUMF2*
ARHGEF4	*FBLN5*	*MAGEF1*	*PSAT1*	*SUSD3*
ARMCX1	*FBXO15*	***MAL***	*PSIP1*	*SYPL1*
ARMCX2	*FCGBP*	*MAN1C1*	*PTPRK*	*TAF4B*
ATG9B	*FHIT*	*MARCKSL1*	*PTTG2*	*TATDN1*
AXIN2	*GCET2*	*MMP11*	*RAB30*	*TBXA2R*
BANF1	*GJB6*	*MPP6*	*RAB33A*	*TCEA3*
BCAS4	*GLS2*	*MRPL52*	*RAB3GAP1*	*TCF7*
BCL9	*GP5*	*MTA3*	*RAB43*	*TGIF2*
BEX1	*GPC2*	*MTUS1*	*RALA*	*THOC3*
BEX2	*GRAP*	*MYB*	*RANBP6*	*THYN1*
BIRC2	*GSTM2*	*N6AMT1*	*RBM11*	*TMEM123*
BZW2	*GTF2IRD2*	*NAP1L3*	*RGMB*	*TMEM69*
CAMSAP1L1	*H2AFY2*	*NDFIP1*	*RPIA*	*TMEM97*
CASP6	*HEMGN*	*NECAP2*	*RPL22*	*TNFRSF10D*
CBR3	*HERC3*	*NELL2*	*SAE1*	*TOP1MT*
CCDC25	*HIRIP3*	*NET1*	*SAP18*	*TSGA10*
CCNG1	*HKDC1*	*NGFRAP1*	*SCML1*	*TSNAX*
CCR7	*HPCAL1*	*NOG*	*SDCCAG8*	*TSPAN3*
CD248	*HSD17B8*	*NOSIP*	*SDK2*	*VIPR1*
CD55	*HSPB1*	*NPM3*	*SELL*	*VNN2*
CDCA7L	*ICOS*	*NRCAM*	*SERPINE2*	*VPS24*
CHI3L2	*IL6ST*	*NSMCE1*	*SERTAD2*	*YPEL2*
CIAPIN1	*IMPDH2*	*NUCB2*	*SETMAR*	*ZNF101*
CITED4	***ITK***	*OR2A9P*	*SFXN4*	*ZNF154*
CLEC11A	*KBTBD11*	*P2RY10*	*SH3YL1*	*ZNF181*
CLN5	*KCNMB4*	*PCSK5*	*SIRPG*	*ZNF200*
CMTM7	*KRT18*	*PDE9A*	*SLC16A10*	*ZNF415*
CNN3	*LAMC3*	*PECAM1*	*SLC22A17*	*ZNF439*
CNOT7	*LDLRAP1*	*PHGDH*	*SLC24A6*	*ZNF671*
COL18A1	*LEF1*	*PIGC*	*SLC25A26*	

Table S7, related to Table 1: Core transcriptional signature of CD161-associated downregulated genes. Leading edge analysis was performed on the GSEA of CD161++ downregulated genes in all T cell subsets. The leading edge gene set for each was compared and a core set of 199 genes identified and listed in alphabetical order. Those referred to in the text are highlighted in bold.

Supplemental Experimental Procedures

Flow cytometry
Antibodies/dyes used were as follows: viability dye Live/Dead fixable-Near-IR (Invitrogen), CD3-PECy7 or-APC, CD8-eFluor450, IL18Rα-PE (eBioscience), CD161-PE or –APC, IFNγ-FITC (Miltenyi Biotec), Vα7.2 –APC, –FITC or –PE, CXCR6-AlexaFluor647, MDR1/CD243-PE, CD3-PECy7, CD8-PE, CD161-BV421 (BioLegend), IFNγ-AlexaFluor 700 or –PerCPCy5.5, RORγt-PE (BD Bioscience), PLZF-APC and CCR6-FITC (R&D Systems), anti-TCRγδ-FITC and –PC5, and T cell receptor Vβ antibodies; Vβ 1, 2, 4, 5.3, 7.2, 8, 9, 11, 13.2, 14, 16, 18, 23-PE, and Vβ 3, 5.1, 5.2, 7.1, 12, 13.1, 13.6, 14, 16, 17, 20, 21.3, 22-FITC (Beckman Coulter). Data were acquired on a MACSQuant (Miltenyi Biotec) or LSRII (BD Bioscience) and analysed using FlowJo (Treestar, Inc.).

CyTOF
After stimulation, cells were stained with fluorescently-tagged primary antibodies for 30min on ice. Cells were then washed and stained with a cocktail of metal-conjugated surface-marker antibodies including secondary antibodies against fluorescent tags (see Table below) for 30min on ice. Cells were then resuspended in 20μM maleimido-mono-amin-DOTA in PBS for 30min on ice for identification of dead cells. Cells were washed extensively and fixed overnight in 2% paraformaldehyde at 4°C. The next day, cells were washed in permeabilisation buffer (eBioscience) and stained with a cocktail of intracellular antibodies on ice for 45min (see Table below). Cells were then washed and labeled for 20min at room temperature with 250nM iridium interchelator (DVS Sciences) suspended in PBS + 2% paraformaldehyde. Cells were extensively washed and resuspended in distilled water before acquisition on the CyTOF in dual-count mode with noise-reduction mode turned off. Analysis was performed on FlowJo software (Treestar, Inc.). For principal component analysis (PCA) live CD8+ T cell events were exported and analysed with scripts written in Matlab. Matlab scripts produced text files which were converted back to FCS files with a custom algorithm written in Java to pdb files read by PyMOL software (DeLano Scientific LLC) to produce quicktime movies.

CD161 Ligation
CD161 ligation was performed using biotin beads from the T cell activation/expansion kit (Miltenyi Biotec) loaded following manufacturer's instructions with the addition of anti-CD161 clone 191B8 (Miltenyi Biotec) or IgG2a (eBioscience), or by a 300 cell line transfected with LLT1 (300-LLT1; kindly provided by G. Freeman). The parental cell line (300-Null) was used as a control. Vα7.2+ cells were enriched using Vα7.2-PE antibody (BioLegend) and anti-PE Microbeads (Miltenyi Biotec), as per manufacturer's instructions.

Base Panel	Atomic Mass
"cell length"	N/A
DNA	191, 193
Live/Dead	103, 115
CD3	QDot = Cd-122 and others
CD3	141
CD8	146
CD4	149
CD13	156
CD33	156
CD19	157
TCRγδ-FITC	162
**Phenotypic/ Functional Markers**	**Atomic Mass**
CD5	143
CCR7	168
CD107a+b	153
CD127	165
CD161-APC	167
CD27	154
CD28	160
CD38	161
CD40L	172
CD45RA	148
CD45RO	144
CD56	159
CD57	115
CD62L	174
CTLA4	145
GM-CSF	147
Granzyme B	151
HLA-DR	171
IFNγ	170
IL-2	166
MIP1β	150
Perforin	176
TNFα	152
